# Cochlear Implantation in Down Syndrome: Functional Outcomes, Challenges, and Management Strategies

**DOI:** 10.3390/audiolres16020044

**Published:** 2026-03-09

**Authors:** David H. Elisha, David H. Cohen, Andrea Monterrubio, Ryan Hossain, Nicholas DiStefano, Rahul Mittal, Adrien A. Eshraghi

**Affiliations:** 1 Hearing Research and Cochlear Implant Laboratory, Department of Otolaryngology, University of Miami Miller School of Medicine, Miami, FL 33136, USA; 2 Faculty of Medicine, Tel Aviv University, Tel Aviv 6997801, Israel; 3 Herbert Wertheim College of Medicine, Florida International University, Miami, FL 33199, USA; 4 Department of Neurological Surgery, University of Miami Miller School of Medicine, Miami, FL 33136, USA; 5 Department of Biomedical Engineering, University of Miami, Coral Gables, Miami, FL 33146, USA

**Keywords:** cochlear implant, Down syndrome, outcomes

## Abstract

**Objective:** The aim was to evaluate cochlear implantation (CI) outcomes in children with Down syndrome (DS) with severe-to-profound sensorineural hearing loss (SNHL), addressing a literature gap and discussing challenges including anatomical abnormalities, cognitive deficits, and Eustachian tube dysfunction. **Data Sources:** Systematic searches were conducted in PubMed, Web of Science, Scopus, and Embase from inception through to June 2025. **Review Methods:** A systematic review adhering to PRISMA guidelines was performed. Included studies reported CI outcomes in DS patients receiving otolaryngologic care for SNHL. Extracted data included findings on ear anatomy, auditory performance, speech/language development, intelligibility, and duration of CI use. **Results:** A total of 149 abstracts were screened, yielding six studies with 26 patients that met the inclusion criteria. The review included pediatric DS patients with documented ages at implantation spanning from 11 months to 17.9 years. CI provided significant benefits for DS patients, including improved audiometric results, enhanced environmental awareness, and psychosocial gains. Optimal outcomes were associated with early implantation, thorough preoperative imaging (CT/MRI), and management of middle ear disease. Variability in outcomes often reflected cognitive limitations and anatomical challenges such as cochlear nerve hypoplasia and Eustachian tube dysfunction. **Conclusions:** CI can significantly improve quality of life and communication in children with DS when tailored to their unique needs. Preoperative imaging is essential to assess candidacy, and middle ear disease should be addressed prior to surgery. Clinicians should counsel families with individualized goals that emphasize functional hearing gains over normative speech benchmarks. Broader adoption of CI in this population may be supported by standardized, population-sensitive outcome measures and future prospective studies.

## 1. Introduction

Down syndrome (DS), resulting from Trisomy 21, affects approximately 1 in 700 live births and presents significant otolaryngologic challenges [1,2,3,4,5,6,7,8,9,10,11,12,13,14,15,16]. Hearing loss occurs in up to 80% of DS children [3,17], predominantly conductive, though sensorineural hearing loss (SNHL) affects 4–20% of cases [3,18,19]. These cases stem from anatomical abnormalities of the temporal bone as well as Eustachian tube dysfunction leading to recurrent otitis media [1,4,6,7,20,21,22].

DS patients experience increasing hearing loss with age, necessitating repeated ventilation tube (VT) placements [23,24,25]. Patients undergo an average of 3.5 VTs over 15 years, with residual tympanic membrane perforations in 17% of cases [25,26], highlighting the additional complications DS patients face following tube placements [27,28]. Hearing aids have been proposed as an alternative to repeated tube placements [13,29,30], and bone-anchored hearing aids (BAHAs) have shown significant quality of life (QoL) improvements with no significant increase in complication rates [31]. More frequent otologic exams and audiologic screenings every 3–6 months are recommended to optimize hearing levels and overall management [25].

**Figure 1:**
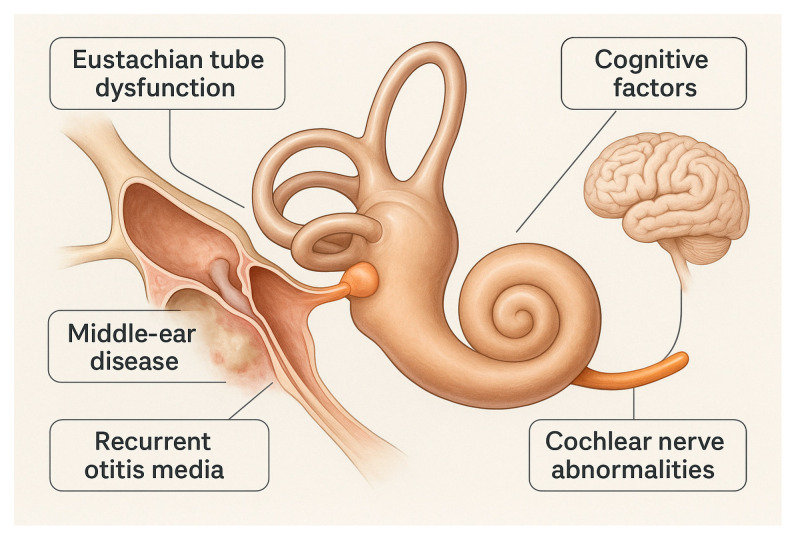
Down syndrome-specific challenges impacting cochlear implantation. Schematic illustration highlighting key anatomical and clinical factors commonly encountered in children with Down syndrome that may influence cochlear implant candidacy and outcomes, including middle ear disease, Eustachian tube dysfunction, cochlear nerve abnormalities, and cognitive considerations. Created in BioRender. https://BioRender.com/7r0imuv (accessed on 15 December 2025).

While cochlear implantation (CI) has become the standard treatment for severe SNHL in the general population [32,33,34,35,36], standardized guidelines for CI in children with developmental disabilities remain undefined [3,37,38,39,40,41,42,43]. DS patients have been underrepresented in CI research due to recurrent otitis media [44,45,46,47,48], concerns about cochlear nerve (CN) absence [49], and cognitive delays potentially impacting outcomes [1,2,3] (Figure 1).

Previous reviews of CI in syndromic or medically complex pediatric populations have included individuals with DS only incidentally and without systematic evaluation of DS-specific candidacy considerations or outcomes. To date, no comprehensive systematic review has focused exclusively on CI in individuals with DS.

This systematic review analyzes the current literature on CI outcomes in DS patients, evaluating the indications, contraindications, benefits, and challenges of using standardized measures such as categories of auditory performance (CAP) and speech intelligibility rating (SIR). We will examine correlations between duration of CI use and functional outcomes, anatomical abnormalities, and broader QoL implications. By synthesizing the current information, we aim to provide evidence-based guidance for CI in DS patients, highlighting potential benefits while acknowledging associated challenges. This review aims to support development of more inclusive CI protocols, potentially expanding access to this intervention for individuals with DS who may be appropriate candidates.

## 2. Methods

This study followed PRISMA guidelines with protocol registered in INPLASY (Registration number: INPLASY202520103). Comprehensive literature searches were performed in four databases (PubMed, Embase, Web of Science, and SCOPUS) from inception through to June 2025. The search strategy employed Medical Subject Heading (MeSH) terms and keywords combined with Boolean operators (AND, OR). The complete search string for PubMed was: (“Cochlear Implants”[MeSH] OR “Cochlear Implant*” OR “CI”) AND (“Down Syndrome”[MeSH] OR “Trisomy 21” OR “Chromosome 21 Trisomy”) AND (“Hearing Loss, Sensorineural”[MeSH] OR “Sensorineural Hearing Loss” OR “SNHL” OR “Deafness”) AND (“Speech Perception”[MeSH] OR “Speech Recognition” OR “Auditory Performance” OR “Speech Development” OR “Language Development”). Similar search strategies were adapted for Embase, Web of Science, and SCOPUS databases, maintaining equivalent search logic across platforms. No language restrictions were applied.

Two reviewers (DC and DE) screened articles using the Covidence platform (Veritas Health Innovation, Melbourne, Australia). Disagreements were resolved by senior authors (AAE and RM). Inclusion criteria encompassed studies examining CI in DS individuals focusing on implantation outcomes, auditory performance, speech/language development, and related clinical considerations. Exclusion criteria included non-DS patients, studies not related to cochlear implantation, review articles, and non-peer-reviewed publications.

Two researchers (DC and DE) utilized The Joanna Briggs Institute (JBI) checklist for critical appraisal. Discrepancies were resolved through discussion and consensus, with senior author (AAE and RM) input when necessary. Data extracted included anatomical abnormalities of the ear, auditory performance, speech/language development and CI duration.

## 3. Results

**Figure 2:**
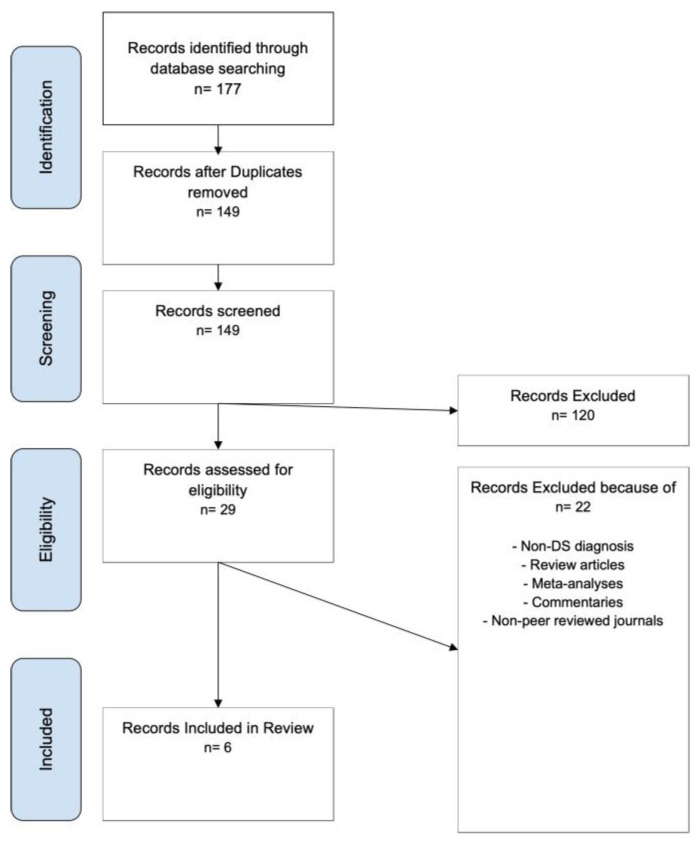
PRISMA flow diagram of study selection for cochlear implantation in Down syndrome.

A total of 177 records were identified. After removing duplicates, 149 records were screened, with 120 excluded based on title and abstract. Twenty-nine full-text articles were assessed, and 22 were excluded due to non-DS diagnoses, review articles, commentaries, or non-peer-reviewed journals. Six studies met the inclusion criteria and were included in the final review. A PRISMA flow diagram is shown in Figure 2.

The six included studies, published between 2010 and 2024, encompassed 26 DS patients who had undergone CI [Table 1 and Supplementary Table S1]. The review included pediatric DS patients with documented ages at implantation spanning from 11 months to 17.9 years. Follow-up duration varied across studies, with CI use documented from 12 months to 19 years post-implantation. The risk-of-bias assessment is presented in Supplementary Figures S1 and S2. Studies were evaluated to be of high methodological quality with low risk, supporting their inclusion.

### 3.1. Patient Characteristics and Preoperative Assessment

A review of the included studies demonstrates that the preoperative evaluation of children with DS largely followed the established protocols used for non-syndromic pediatric cochlear implant candidates, although several DS-specific considerations were emphasized. All studies that described preoperative assessment reported the routine use of high-resolution computed tomography and magnetic resonance imaging, reflecting the increased prevalence of temporal bone malformations, narrowed internal auditory canals, and cochlear nerve hypoplasia in this population. As a result, imaging played a more central role in determining candidacy than is typically required for children without congenital syndromes.

The studies also highlighted the necessity for repeated otologic examinations and management of chronic middle ear disease prior to implantation, since persistent effusion and recurrent acute otitis media are more frequent in children with DS and can obscure accurate assessment of sensorineural hearing thresholds. No study reported the use of electrophysiological assessments such as electrically evoked compound action potentials, electrically evoked auditory brainstem responses, or cortical auditory evoked responses in the preoperative evaluation. Likewise, none of the included studies described modifications to subjective behavioral testing or changes to the timing of audiologic assessments beyond those dictated by the delayed diagnosis that often accompanies coexisting conductive pathology.

Overall, the available evidence suggests that while the core components of cochlear implant candidacy assessment were consistent with general pediatric practice, the DS population requires more intensive imaging and clinical surveillance, whereas advanced electrophysiological measures were not incorporated into the preoperative protocol in any of the reviewed reports.

### 3.2. Anatomical Abnormalities and Middle Ear Pathology

Anatomical abnormalities were systematically documented in four studies, revealing a high prevalence of both middle and inner ear malformations in the DS population undergoing CI evaluation. Heldahl et al. evaluated eight DS patients who received CI [37]. Three patients had no middle ear pathology, while five had otitis media with effusion (OME). Additional findings included cholesteatoma, retraction, and recurrent acute otitis media (rAOM) with left-side perforation. Based on CT and MRI, all but one patient exhibited ear malformations, including deformities of the semicircular canal (SCC), cochlea, internal auditory canal (IAC), vestibular organ, and external auditory canal (EAC), as well as CN hypoplasia [37].

Hans et al. described middle ear disease and imaging abnormalities in four patients [2]. All patients had severe-to-profound SNHL and underwent comprehensive preoperative assessments consisting of imaging and evaluations of cognitive and communication abilities. Middle ear disease included previous VT insertions, middle ear effusions, and AOM. Regarding imaging abnormalities, two patients had none, while one patient exhibited middle and inner ear characteristics of DS based on CT and another had absence of CN on the right side based on MRI [2].

Claros et al. completed a retrospective analysis comparing nine children with DS to 220 non-syndromic pediatric patients divided into four categories depending on the age of first implantation [3]. Anatomical abnormalities were more prevalent in the DS population compared to the non-syndromic population. Based on radiological evaluation of 90% of the DS cohort, abnormalities included narrowed EAC with residual cerumen (40%); poor mastoid pneumatization and small middle ear cavity (30%); incomplete partition of the cochlea type 1 and 2 (IP-1 and IP-2) (20%); and CN absence (unilateral and bilateral), enlarged vestibular aqueduct (EVA), semicircular canal dehiscence (SSCD), and narrowed IAC (10%) [3].

Lorente-Piera et al. conducted a retrospective analysis of 16 pediatric patients with DS utilizing high-resolution computed tomography (CT) and magnetic resonance imaging (MRI) to evaluate otologic anatomy and pathology [4]. Importantly, the three patients who underwent CI exhibited congenital inner ear abnormalities, including type II cochlear hypoplasia in two cases and CN hypoplasia in one [4].

Phelan et al. conducted a retrospective case series of three children with DS to evaluate CI candidacy [50]. One child proceeded to implantation at 4 years and 9 months of age following delayed diagnosis of profound bilateral sensorineural hearing loss. This patient had multiple middle and inner ear abnormalities, including a sclerotic mastoid, granulation tissue, absent ossicular elements (incus and stapes), and dehiscence of the facial nerve at the horizontal segment and second genu. Imaging revealed hypoplasia of the CN and stenosis of the internal auditory canal contralaterally, though the implanted side demonstrated normal cochlear anatomy, permitting full electrode insertion without complications [50].

### 3.3. Functional Outcomes

#### 3.3.1. Categories of Auditory Performance (CAP) Score

Four studies incorporated CAP assessment. CAP scores range from 0 to 7, assessing auditory performance from no sound awareness to telephone use [2,37,52,53].

Heldahl et al. conducted a retrospective study of eight patients, reporting CAP scores at 3 months, 12 months, and the last follow-up [37]. All patients achieved a minimum score of 2; with individual patients reaching higher scores over time ranging from 2–3 to 5–6 after periods of 3.5 to 12 years post-implantation. These scores represent progression from responding to speech sound to understanding simple conversations without lipreading [37].

Hans et al. conducted a survey of British Cochlear Implant Group programs, identifying four children who underwent CI [2]. CAP scores varied depending on the time after implantation, ranging from 1 to 4–5 over periods of 12 to 50 months post-implantation. All four patients demonstrated consistent device usage throughout this follow-up period [2].

Phelan et al. reported one patient with DS implanted at 4 years and 9 months, documenting a CAP score of 0 pre-implant, which improved to 4 at both six months post-operatively and at last follow-up [50]. Despite the presence of significant anatomical abnormalities and developmental delay, the patient demonstrated consistent device use and functional auditory gains, highlighting the potential benefit of CI even in complex cases [50].

Claros et al. conducted a comparative retrospective analysis of nine DS patients who underwent CI, compared to 220 non-syndromic patients [3]. The DS group demonstrated significantly lower scores (mean of 4.4, SD 0.9, *p* = 0.02) compared to the non-syndromic group. Further analysis revealed that DS patients implanted before the age of 6 achieved better and faster outcomes, with significantly higher CAP scores after 5 years (*p* < 0.05). However, CAP score improvement plateaued for both groups after 5 years post-implantation (*p* > 0.05). Long-term follow-up in this cohort ranged from 10 to 19 years, representing the most extensive duration of CI use among the included studies [3].

#### 3.3.2. Speech Intelligibility Rating (SIR) Score

Three studies analyzed SIR scores as a measure of speech intelligibility outcomes. SIR scores measure speech intelligibility [2,37,54]. Heldahl et al. evaluated SIR scores in eight children who underwent bilateral CI [37]. Most patients demonstrated limited intelligibility of spoken language: five children attained an SIR score of 1, indicating pre-recognizable words, and two reached a score of 1–2. Only one child achieved an SIR score of 3, reflecting connected speech intelligible to an experienced listener. These outcomes were documented over follow-up periods ranging from 3.5 to 12 years [37].

Based on survey data, Hans et al. reported that all four patients had an SIR score of 1 after 12 to 50 months of CI use, indicating that their spoken language remained at the level of pre-recognizable words [2]. Despite consistent implant use in some cases and gradual improvements in auditory responsiveness, none of the patients developed intelligible connected speech [2].

Claros et al. found that SIR scores were significantly lower in the DS population, with a mean SIR score of 3.2 (SD 0.7, *p* = 0.03, Mann–Whitney = 579) [3]. When comparing implantation before and after age 6, both groups showed significant improvement in the first 5 years post-implantation (*p* < 0.001), followed by stabilization after 5 years (*p* > 0.05). However, patients implanted before the age of 6 achieved higher scores at each measured period [3].

#### 3.3.3. Audiometric Outcomes and Additional Measures

Lorente-Piera et al. reported audiometric results for three DS CI patients with the follow-up averaging 7.07 ± 2.04 h per day of device usage [4]. All three patients initially demonstrated severe-to-profound SNHL, with preimplantation thresholds exceeding 100 dB HL. One year after implantation, there was marked improvement in hearing thresholds, reducing to the mild-to-moderate range. Specifically, post-implantation thresholds improved to approximately 35 to 50 dB HL. The gain in hearing, measured as the difference between preimplantation and post-implantation thresholds, was substantial across all patients. Patient 1 showed a gain of approximately 70 dB, Patient 2 improved by around 60 dB, and Patient 3 achieved a gain close to 80 dB [4].

Broomfield et al. analyzed 38 children with syndromic deafness, including one DS child [51]. SRS classifies speech perception ability on a seven-grade scale (0–6), ranging from no speech perception to open-set word recognition [55]. The DS child achieved a score of 4, demonstrating identification of words differing by one vowel [51].

Claros et al. evaluated MAIS and MUSS for auditory performance and speech and language development [3]. The MAIS score was not compared between the DS population and non-syndromic population, as scores for the latter were unavailable. However, MAIS scores were compared between patients implanted before and after the age of 6. The questionnaire-based measure yielded an average score of 76.9% (SD 0.1). The younger group demonstrated significantly higher and faster recognition and response to sound (*p* < 0.001), but scores in both groups plateaued 10 years post-implantation (*p* > 0.05).

MUSS, another questionnaire-based measure, was compared between the DS and non-syndromic population, with DS patients scoring significantly lower (60%, SD 0.1, *p* = 0.03, Mann–Whitney = 576.5). When comparing implantation before vs. after age 6, the younger group initially exhibited lower speech performance but outperformed the older group between 1 and 5 years post-implantation. Both groups showed significant improvement up to 10 years (*p* < 0.001), after which scores stabilized (*p* > 0.05) [3].

## 4. Discussion

This systematic review synthesized evidence from six studies encompassing 26 pediatric patients with DS who underwent CI. All studies demonstrated auditory benefit following implantation, though considerable heterogeneity in patient characteristics, anatomical findings, outcome measures, and follow-up durations limited direct comparisons. Despite these limitations, the findings support the feasibility and value of CI in appropriately selected DS children with severe-to-profound SNHL, provided that comprehensive preoperative evaluation is conducted and realistic expectations are established.

### 4.1. Anatomical Considerations

Anatomical abnormalities in DS children present CI challenges and predispose to hearing loss through narrower Eustachian tubes, ear canals, and cochlear malformations [14]. CT and MRI scans commonly assess the extent of temporal bone, external, middle and inner ear malformations. The studies included in this review consistently identified various anatomical abnormalities in DS patients, some of which directly influence CI eligibility and outcomes. The most frequently observed abnormalities include malformations of the SCC, cochlea, CN, IAC, EAC, and vestibular aqueducts [6,37].

One study identified malformed bone islands of lateral SCC as most common among DS children, whereas IAC narrowing had the highest odds ratio for SNHL [6]. However, while malformations are common in DS children, not all are associated with SNHL development [6]. The only noted contraindication to CI in DS children is a narrow IAC or cochlear canal, as these patients tend to have the poorest post-implantation outcomes, possibly due to potential CN absence or deficiency [6,56]. Intact CN presence is essential for CI, evidenced by patients with bilateral absence disqualifying implantation, while unilateral absence limits CI to the intact side [2,3].

For DS patients presenting with hearing loss, inner ear dysplasia evaluation should be conducted, as it is common in this population [57]. A comparative study between DS and non-syndromic, normal-hearing individuals found that DS patients exhibited greater prevalence of hypoplastic cochlea, smaller CN canals, smaller IACs, hypoplastic lateral semicircular canals (<3 mm), hypoplastic vestibules, and enlarged vestibular aqueducts [58]. This finding aligns with the prior literature and reinforces individualized assessment needs in DS patients, as certain structural differences may affect electrode placement and post-implantation hearing outcomes.

The findings from this review emphasize that anatomical abnormalities, while common, should not automatically disqualify DS children from CI consideration. Phelan et al. demonstrated that even patients with significant anatomical complexity, including absent ossicular elements and facial nerve dehiscence, could achieve successful electrode insertion and functional outcomes when cochlear anatomy on the implanted side was preserved [50]. This suggests that the critical determinant is not the presence of any abnormality, but rather the specific preservation of structures essential for CI function: an intact cochlear nerve, patent cochlea capable of accepting an electrode array, and manageable middle ear pathology.

While anatomical abnormalities are common in DS patients, they do not necessarily exclude them from benefiting from CI. Instead, high-resolution imaging combined with functional assessments, such as repeated otologic exams, debridements, and audiologic evaluations, is critical to optimizing patient selection and surgical planning.

### 4.2. Functional Outcomes and Cognitive Considerations

Studies suggest that CI improves development and QoL in DS children, although outcomes may differ compared to non-syndromic hearing-impaired children. These differences are largely attributed to cognitive and intellectual impairments inherent to DS, affecting language development and post-implant performance. While DS children may demonstrate slower progress and comparatively lower speech and language outcomes, objective auditory improvements following CI have been observed [2].

A comparative study by Claros et al. provided the most robust evidence of this outcome disparity, demonstrating that DS patients achieved significantly lower CAP scores (mean 4.4, *p* = 0.02), SIR scores (mean 3.2, *p* = 0.03), and MUSS scores (60%, *p* = 0.03) compared to children without syndromic diagnoses [3]. However, it is important to contextualize these findings: a CAP score of 4 represents the ability to discriminate some speech sounds without lipreading, and an SIR score of 3 indicates connected speech intelligible to an experienced listener, both representing functional communication abilities. Thus, while outcomes may be quantitatively lower, they still reflect clinically meaningful gains.

The outcome heterogeneity across the included studies likely reflects differences in multiple unmeasured factors. Hans et al. reported that all four DS patients achieved an SIR score of only 1 (pre-recognizable words) despite consistent device usage over 12–50 months [2], while Heldahl et al. documented one patient reaching an SIR score of 3 and several achieving CAP scores of 5–6 over longer follow-up periods of 3.5–12 years [37]. This variability may reflect differences in follow-up duration, age at implantation, individual cognitive capacity, intensity of auditory habilitation, family engagement, or other confounding factors not systematically assessed in these studies.

The outcomes observed in DS children can be contextualized within the broader landscape of syndromic hearing loss. Saleeb et al. reported successful CI outcomes in 45 syndromic children despite inner ear malformations in 20% of cases [42]. Syndromes without cognitive impairment (Jervell and Lange-Nielsen, Waardenburg) achieved outcomes comparable to non-syndromic patients, while syndromes with structural abnormalities (CHARGE, Branchio-oto-renal) still benefited from CI with individualized surgical approaches. This suggests anatomical complexity is manageable, whereas cognitive impairment more significantly affects speech and language development. DS is distinguished by a combination of universal intellectual disability and chronic middle ear disease, necessitating individualized assessment rather than categorical exclusion.

Given the unique cognitive limitations in this population, outcome measures should be tailored to reflect realistic goals for DS children, rather than benchmarking progress against typical CI recipients. Rather than focusing on achieving near-normal spoken language skills, the primary CI objective in DS patients should emphasize enhancing stimulus responses and optimizing communication abilities within their cognitive capacity [37].

Hearing loss, especially severe-to-profound hearing loss, has a well-documented detrimental impact on speech, language, cognition, and behavior in children. This effect is even more pronounced in DS patients who already experience baseline intellectual disabilities [59]. Furthermore, the presence of hearing loss in DS children can complicate cognitive assessments, making developmental progress evaluation challenging [60]. Therefore, early identification and intervention with CI play crucial roles in maximizing developmental outcomes and improving overall QoL in this population.

### 4.3. Age at Implantation and Timing Considerations

The age of CI in children with DS has been a key area of interest in evaluating long-term outcomes and effectiveness. Claros et al. compared outcomes between those implanted before and after six years of age, finding that while testing results in initial evaluation of the younger group were lower, their long-term outcomes, including communication skills, speech, and vocalization, were superior compared to the older age group [3]. Similarly, Phelan et al. noted that delays in implantation may negatively impact CI outcomes [50]. These results align with the broader literature emphasizing the sensitive period for central auditory development, which is critical for maximizing oral speech and language acquisition. Studies have shown that children implanted before four years old outperform those implanted after seven years old in sentence recognition tasks [61]. Additionally, early auditory stimulation may contribute to auditory neural survival and improved functionality of auditory centers [62]. Collectively, these findings suggest that age at implantation is a key predictor of CI success in DS children, with earlier implantation yielding greater benefits.

However, a challenge in determining CI candidacy in DS children lies in the criteria requiring severe-to-profound SNHL. DS patients with hearing problems are less likely to receive cochlear implants, as conductive hearing loss (CHL) is more common in this population than SNHL. While SNHL prevalence tends to increase with age in DS patients, this pattern contradicts the ideal timeframe for early implantation. The pathogenesis of progressive SNHL in DS is not well understood, though chronic middle ear disease has been proposed as a contributing factor [18,28]. It is also observed that individuals with DS experience age-related hearing loss 20–30 years earlier than the general population [63].

This creates a clinical paradox unique to the DS population: the optimal developmental window for CI (early childhood) often precedes the development of severe-to-profound SNHL in many DS children, yet delaying implantation until audiometric criteria are definitively met may reduce potential benefit from the intervention. The wide variability in age at implantation observed across studies in this review reflects this clinical challenge. The superior outcomes observed in children implanted before age six in the Claros study [3] support aggressive pursuit of early implantation when candidacy criteria are met, rather than adopting a conservative “wait and see” approach that may sacrifice critical developmental opportunities.

Rather than reinforcing early CI in all DS patients, these findings highlight the importance of early diagnosis and management of underlying middle ear disease to potentially prevent or delay progression of SNHL, ensuring that CI candidates are identified and treated at the most beneficial time.

### 4.4. Middle Ear Management and Practical Considerations

Eustachian tube (ET) dysfunction is very common in DS children, contributing to higher middle ear disease incidence. In DS, the ET is often malformed and narrower, predisposing them to middle ear effusion and chronic middle ear dysfunction [12,48,61,62,63]. Additionally, a thinner and reduced cartilaginous cell density in the ET wall increases its susceptibility to collapse, leading to persistent otitis media. Recurrent middle ear infections in DS complicate hearing loss management, as conductive hearing loss (CHL) may develop and interfere with optimal auditory outcomes [14]. If left untreated, chronic middle ear disease can progress to cholesteatoma formation, adding complexity to hearing management in this population [64].

For DS patients with otitis media with effusion (OME), the standard treatment involves VT placement. However, intervention efficacy in the DS population may be limited due to higher infection recurrence, minimal long-term hearing improvement, and increased risk of tympanic membrane (TM) perforations. The latter is attributed to thinner ear drums with deficient lamina propria, making DS patients more vulnerable to chronic perforation post-VT placement. Given these findings, conservative OME management should be prioritized before considering repeat VT insertions in DS children [28].

Despite these challenges, middle ear management remains essential for CI candidacy. Claros et al. reported that 40% of the DS children who underwent CI had prior VT insertions, reflecting the high prevalence of middle ear disease in this population and underscoring the clinical reality that many DS children require middle ear intervention as part of their pathway to implantation [3].

Similarly, Luntz et al. evaluated CI outcomes in otitis media-prone patients, comparing them to a non-otitis-media group [65]. Their study implemented a preoperative management protocol that emphasized VT placement to treat otitis media rather than relying solely on conservative treatment. Once CI candidacy was confirmed, VTs were placed in patients presenting with AOM or secretory otitis media, irrespective of tympanic membrane status. Following a two-week period, otoscopic re-evaluation was conducted to assess middle ear status. If the VT was dry (i.e., no otorrhea), CI proceeded as planned. However, if the VT remained draining, a secondary management protocol was initiated. This included obtaining cultures, administering targeted antibiotic therapy, and considering VT removal and reinsertion or additional surgical interventions. CI was deferred until the middle ear was dry and clinically stable [66]. Although the study did not exclusively examine DS patients, it underscored the importance of aggressive otitis media management prior to CI. Notably, intraoperative findings in otitis media-prone CI patients often revealed round window obstruction due to inflamed mucosa, which required additional surgical intervention but did not pose significant complications during CI. Additionally, VTs were retained intraoperatively, with postoperative AOM cases managed conservatively with antibiotics. In rare cases, chronic otitis media post-CI required re-insertion of a VT. The study suggested that aggressive preoperative otitis media treatment contributed to a lower incidence of postoperative infections, reinforcing the feasibility of CI in DS despite their predisposition to recurrent acute otitis media [rAOM] [66].

Overall, ET malformations and their associated complications should be a major consideration in determining CI candidacy in DS children. Preoperative management is crucial, ensuring that middle ear disease is optimally treated before implantation. Additionally, close postoperative monitoring is essential to promptly address rAOM and prevent long-term complications, ultimately optimizing hearing outcomes in DS patients undergoing CI. Key DS-specific clinical considerations for CI are summarized in Table 2.

### 4.5. Limitations

Children with DS are significantly underrepresented in CI research [1], leading to limited data on CI outcomes in this population. This systematic review, encompassing only six studies with 26 patients in total (1–9 patients per study), highlights a critical limitation that restricts generalizability. The small sample prevents definitive conclusions about optimal patient selection, timing, or expected outcomes and precludes meaningful subgroup analyses based on cognitive function, anatomical phenotypes, or pre-implant communication modalities.

The included studies exhibited substantial heterogeneity. The age at implantation ranged from 11 months to 17.9 years, follow-up from 12 months to 19 years, and outcome measures were inconsistently applied (CAP, SIR, MAIS, MUSS, SRS, audiometric thresholds). All studies were retrospective case series. This heterogeneity prevents meta-analysis and limits firm conclusions about success predictors. Most critically, none formally assessed cognitive function despite its clear relevance to language outcomes, preventing stratification by intellectual disability severity, a question of direct clinical relevance for family counseling.

Additional limitations include lack of long-term follow-up (restricting evaluation of sustained benefits and complications), inconsistent surgical detail reporting (limiting understanding of approaches for anatomical variations), and absence of validated QoL instruments. While no studies reported surgical complications, common anatomical abnormalities and chronic infections in DS could contribute to surgical challenges and variable outcomes. Future research should incorporate validated pediatric QoL measures accounting for developmental heterogeneity in DS, providing insight beyond auditory performance metrics alone.

Despite these substantial limitations, consistent patterns emerged. All studies reported auditory benefit following CI, whether measured by audiometry, structured scales, or qualitative descriptions. No study reported CI as ineffective or harmful in DS children, though the benefit magnitude varied. The age–outcome relationship observed by Claros et al., with earlier implantation associated with superior outcomes, aligns with the broader pediatric CI literature, suggesting timing principles applicable to non-syndromic children may extend to DS.

Overall, primary limitations stem from small sample sizes, inconsistent outcome measures, lack of long-term follow up and QoL measures, and unique DS population factors. Addressing these limitations is essential for developing evidence-based CI guidelines in DS children.

## 5. Conclusions and Future Directions

This systematic review supports CI’s positive impact in DS, suggesting improvements in auditory performance, communication, psychosocial development, and QoL. While DS children may show slower progress compared to non-syndromic patients, CI benefits align with their cognitive abilities, challenging the notion that intellectual disability is a contraindication [2,37,50].

Findings highlight the importance of early implantation, likely due to a sensitive period for auditory plasticity. However, given the high middle ear disease prevalence and anatomical variations in DS, preoperative imaging and middle ear management are essential for surgical planning and post-implant success. Additionally, long-term follow-up and rehabilitation remain critical for addressing post-CI complications such as AOM [3,37].

Future research should focus on standardizing CI outcome measures for DS and incorporating population-specific benchmarks such as environmental sound recognition and functional hearing improvements, rather than relying solely on spoken language proficiency. Further studies are also needed to explore the progression of SNHL in DS and investigate preventive interventions to preserve hearing [4,37].

## Figures and Tables

**Table 1:** Patient characteristics and cochlear implantation outcomes in Down syndrome.

Study	N	Median Age at CI	Anatomical Abnormalities	Auditory Performance	Speech Outcomes	Follow-Up Duration	Complications
Hans (2010) [2]	4	39 months (25–46)	Middle ear disease (75%); CN absence (25%)	CAP scores 1–5	SIR scores 1–2	12–50 months	None
Claros (2019) [3]	9	78 months (21–215)	Narrowed EAC (40%); mastoid hypoplasia (30%); CN absence (20%); cochlear malformation (20%)	CAP: 6 (early) vs. 3.5 (late); MAIS: 77%	SIR: 4 (early) vs. 2.5 (late); MUSS: 68% (early) vs. 51% (late)	10–19 years	Device failure (*n* = 1)
Heldahl (2019) [37]	8	29 months (11–83)	CN/SSC hypoplasia (63%); stenotic canals (25%)	CAP: 1–4 (3 mo) → 2–6 (final)	SIR: 1–3 (final)	NR	Cholesteatoma with explant (*n* = 1)
Lorente-Piera (2024) [4]	3	8.4 ± 3.0 years	Cochlear hypoplasia (67%); CN hypoplasia (33%)	Threshold gain: 74 dB	NR	NR	None
Phelan (2016) [50]	1	4.8 years	CN hypoplasia; ossicular anomalies	CAP: 4 (6 mo)	NR	6 months	None
Broomfield (2013) [51]	1	3.3 years	NR	NR	SRS: 4	NR	None

**Table 2:** Key clinical considerations for cochlear implantation in children with Down syndrome.

Clinical Domain	Down Syndrome-Specific Considerations
Anatomical Abnormalities	High prevalence of CN hypoplasia/aplasia, narrowed IAC, and inner ear malformations (hypoplastic cochlea, incomplete partitions, SCC anomalies, enlarged vestibular aqueduct). CN deficiency is a frequent candidacy barrier.
Middle Ear Disease	Very high prevalence of chronic OME, recurrent AOM, cholesteatoma, and persistent TM perforations. ET anatomically small and collapsible, leading to recurrent effusions. Mastoid often sclerotic with small middle ear cavity. Multiple VT placements common with higher complication rates.
Surgical Planning	Higher likelihood of facial nerve dehiscence, ossicular anomalies, obliterated mastoid, and round window obstruction. Staged surgery may be needed for cholesteatoma. Requires additional preoperative planning and intraoperative flexibility.
Audiologic Assessment Challenges	Behavioral audiometry often unreliable; sedated ABR frequently required. Mixed hearing losses common, complicating SNHL determination and delaying candidacy evaluation.
Cognitive and Developmental Impact	Intellectual disability limits speech/language development. Progress slower than non-syndromic children; some require caregiver support for consistent device use. Goals should emphasize functional hearing and environmental awareness over age-typical speech.
Timing, Referral and Postoperative Care	Optimal implantation before age 6, but progressive SNHL may delay candidacy. DS children often under-referred. Post-operatively requires close monitoring for infections, middle ear dysfunction, and mapping adjustments; AAC may remain important.

## Data Availability

No new data were created or analyzed in this study. Data sharing is not applicable to this article.

## Supplementary Materials

The following supporting information can be downloaded at: https://www.mdpi.com/article/10.3390/audiolres16020044/s1. Table S1. Summary of patient characteristics, anatomical findings, and outcomes following cochlear implantation in individuals with Down syndrome. The table summarizes individual and aggregated data from studies reporting cochlear implantation in patients with Down syndrome (DS). Information includes the number of patients (N), middle ear pathology, anatomical malformations (e.g., inner ear abnormalities, cochlear nerve hypoplasia or aplasia), age at implantation, and post-implantation outcomes. Reported outcome measures include CAP (Categories of Auditory Performance), SIR (Speech Intelligibility Rating), MUSS (Meaningful Use of Speech Scale), MAIS/IT-MAIS, and Geers and Moog Speech Reception Score (SRS), where available. Duration of cochlear implant use and reported surgical complications are also indicated. NR = Not reported. Figure S1. Risk of bias assessment for case series using the Joanna Briggs Institute (JBI) checklist. Most domains were rated as low risk, with some concerns related to participant inclusion and reporting. Figure S2. Risk of bias assessment for cohort studies using the Joanna Briggs Institute (JBI) checklist. Most domains were rated as low risk, with limited unclear or unreported items related to confounding and follow-up.
