# Supplementary material for: Cochlear Implantation in Down Syndrome: Functional Outcomes, Challenges, and Management Strategies
Source: Audiol Res. 2026 Mar 9;16(2):44. doi: 10.3390/audiolres16020044 (PMC13010643; doi:10.3390/audiolres16020044)

Supplementary Figures. Risk of Bias Assessment. Risk of bias was assessed using the Joanna Briggs Institute (JBI) Critical Appraisal Tools, with the checklist selected according to study design. Each domain was rated as Low risk, High risk, Some concerns, or No information. Disagreements were resolved by consensus

Supplementary Figure S1. Risk of Bias for Case Series: Risk of bias assessment for four case series using the Joanna Briggs Institute (JBI) checklist. Most domains were rated as low risk, with some concerns related to participant inclusion and reporting.

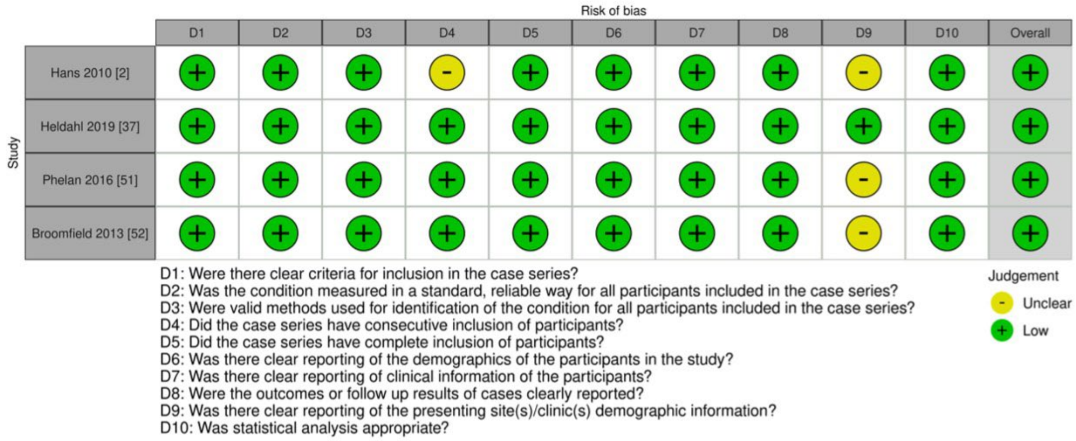

Supplementary Figure S2. Risk of Bias for Cohort Studies: Risk of bias assessment for two cohort studies using the Joanna Briggs Institute (JBI) checklist. Most domains were rated as low risk, with limited unclear or unreported items related to confounding and follow up.

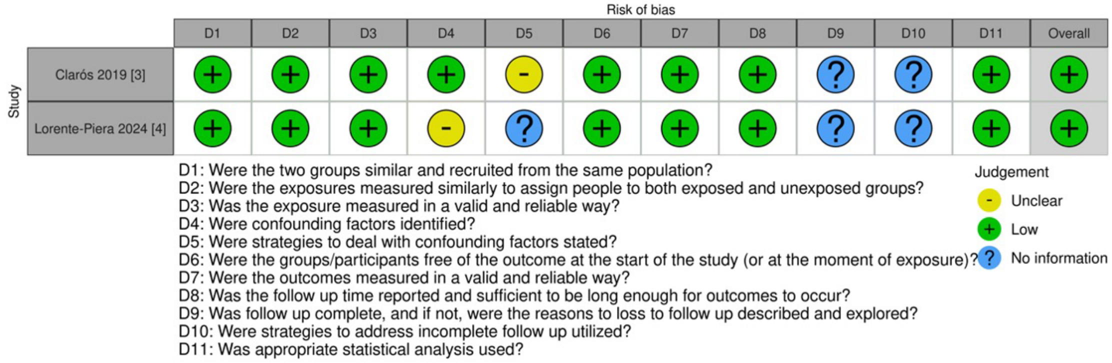

Supplement: Supplementary file 1 [file audiolres-16-00044-s001.zip › Supplementary Figures S1and S2 Risk of Bias 02032026.pdf]
