# Supplementary material for: Cochlear Implantation in Down Syndrome: Functional Outcomes, Challenges, and Management Strategies
Source: Audiol Res. 2026 Mar 9;16(2):44. doi: 10.3390/audiolres16020044 (PMC13010643; doi:10.3390/audiolres16020044)
Supplement: Supplementary file 1 [file audiolres-16-00044-s001.zip › Supplementary Table S1.pdf]

Supplementary Table S1. Summary of Patient Characteristics, Anatomical Findings, and Outcomes Following Cochlear Implantation in Individuals with Down Syndrome

This table summarizes individual and aggregated data from studies reporting on cochlear implantation in patients with Down syndrome (DS). Information includes the number of patients (N), middle ear pathology, anatomical malformations (e.g., inner ear abnormalities, cochlear nerve hypoplasia or aplasia), age at implantation, and post-implantation outcomes. Reported outcome measures include CAP (Categories of Auditory Performance), SIR (Speech Intelligibility Rating), MUSS (Meaningful Use of Speech Scale), MAIS/IT-MAIS, and Geers and Moog Speech Reception Score (SRS), as available. Duration of cochlear implant use and reported surgical complications are also noted where applicable. NR = Not reported.

Author	N (DS with CI)	Patient Characteristics			Outcomes
		Middle Ear Pathology:	Anatomical Abnormalities / Malformations:	Age of Implantation	
Hans (2010) [2]	4	1. 2 previous ventilation tube insertions<br>2. Previous ventilation tube insertion<br>3. Middle ear effusions, Absence CN VIII on R on MRI<br>4. Acute Otitis Media during assessment process	1. Prematurity, cardiac anomaly requiring surgery, middle and inner ear characteristic of DS<br>2. None<br>3. Neo-natal jaundice, gastrointestinal abnormalities<br>4. None	1. 46 months<br>2. 44 months<br>3. 25 months<br>4. 39 months	CAP score:<br>1. 2<br>2. 1<br>3. 2-3<br>4. 4-5<br><br>SIR score:<br>1. 2<br>2. 1<br>3. 1<br>4. 1<br><br>Duration of implant use:<br>1. 12 months<br>2. 20 months<br>3. 36 months<br>4. 50 months

Claros (2019) [3]	9	NR	Narrowed external auditory canal with residual cerumen (40%), poor pneumatization of the mastoid (30%), small cavity of the middle ear (30%), cochlear nerve absence (unilateral 10%; bilateral 10%), enlarged vestibular aqueduct (10%), semicircular canal dehiscence (10%), incomplete partition of the cochlea type 1 and 2 (20%), narrowed internal auditory canal (10%)	1. 82 months<br>2. 65 months<br>3. 54 months<br>4. 127 months at first, 169 months at second<br>5. 21 months<br>6. 70 months<br>7. 78 months<br>8. 102 months<br>9. 79 months at first, 215 months at second	CAP score average by age of implantation:<br>- 0-3 yr: 6<br>- 4-5 yr: 4.7<br>- 6-7 yr: 4.3<br>- 8-17 yr: 3.5<br><br>SIR score average by age of implantation:<br>- 0-3 yr: 4<br>- 4-5 yr: 3.3<br>- 6-7 yr: 3.3<br>- 8-17 yr: 2.5<br><br>MUSS (%) average by age of implantation:<br>- 0-3 yr: 68.2%<br>- 4-5 yr: 62.9%<br>- 6-7 yr: 59.9%<br>- 8-17 yr: 51.1%<br><br>MAIS/IT-MAIS average score:<br>76.9%<br><br>Duration of CI use (yrs) :<br>1. 14<br>2. 12<br>3. 13<br>4. 19<br>5. 13<br>6. 12<br>7. 12<br>8. 10<br>9. 16<br><br>*Surgical Complication:<br>Pt #4 re-implantation - due to device failure

Lorente-Piera (2024) [4]	3	- Acute otitis media (31.25%), seromucosal otitis media (31.25%), chronic otitis media (12.50%), transtympanic ventilation tubes (56.25%), adenotonsillectomy for obstructive sleep apnea syndrome (25%), Myringoplasty (25%), Canaloplasty for cholesteatoma (6.25%), cochlear implantation for severe-to-profound hearing loss (18.75%)<br>- CI only: Severe-to-profound hearing loss (18.75%), 2/3 with type II cochlear hypoplasia, 1/3 with cochlear nerve hypoplasia	- Serous otitis media (31.25%), acute otitis media (31.25%), keratosis obturans (6.25%), adenotonsillar hypertrophy	8.43 +/- 2.95 (Median)	Mean Mid-tonal threshold pre-treatment: 113.35 dB<br><br>Mean Mid-tonal threshold post-treatment: 39.20 dB<br><br>Mean Mid-tonal threshold gain: 74.15 dB
Broomfield (2013) [51]	1	Deafness	NR	Median age of implantation: 3.3 years	Geers and Moog speech reception score (SRS): 4
Heldahl (2019) [37]	8	1. OME cholesteatoma right middle ear 9 years after CI, right CI explanted at cholesteatoma surgery<br>2. None<br>3. Retraction and OME<br>4. OME<br>5. OME, rAOM, perforation left side<br>6. OME<br>7. None<br>8. None	1. Slight deformity of SSC BIL, slightly narrow cochlear BIL<br>2. None<br>3. Slight apical deformity of cochlear BIL, Hypoplasia of right CN<br>4. Slightly narrow IAC right side<br>5. Slightly hypoplastic vestibular organ<br>6. Hypoplastic lateral SSC BIL, widened IAC BIL<br>7. Hypoplastic posterior SSC BIL, BIL stenotic EAC and slight middle ear malformations, hypoplastic cerebellum and vermis<br>8. Hypoplasia of right CN	1. 45 months at first, 83 months at second<br>2. 34 months for both<br>3. 21 months at first, 27 months at second<br>4. 21 months for both<br>5. 64 months at first, 71 months at second<br>6. 11 months at first, 15 months at second<br>7. 23 months at first, 44 months at second<br>8. 29 months for both	CAP score at 3 months; 12 months; last follow up:<br>1. 1;2;2-3<br>2. 2;4;5<br>3. 2;2;3<br>4. 2;3;4<br>5. 2;2;5<br>6. 3-4;4;5-6<br>7. 2;2;5<br>8. 1;2-3;3-4<br><br>SIR score:<br>1. 1<br>2. 1<br>3. 1-2<br>4. 1<br>5. 1-2<br>6. 3<br>7. 1<br>8. 1

					*Pt 1 developed pain and discharge after cholesteatoma removal (9 years after CI) which led to the explant of CI
Phelan (2016) [54]	1	1. OME, Sclerotic mastoid, Granulation tissue in the mastoid and mesotympanum, Absent long process of the incus and absent stapes suprastructure, Dehiscent facial nerve at the horizontal portion and second genu	1. Stenotic internal auditory canal (contralateral side), Hypoplastic cochlear nerve (contralateral side), Normal inner ear anatomy on the implanted (left) side	1. 4 years and 9 months	CAP score at 6 months post-op and at last follow-up: 4

NR = Not reported
